# Editorial: Advances in Metabolic Mechanisms of Aging and Its Related Diseases

**DOI:** 10.3389/fphys.2020.594974

**Published:** 2020-10-09

**Authors:** Daniele Lettieri-Barbato, Natascia Ventura, Raffaella Faraonio, Katia Aquilano

**Affiliations:** ^1^Department of Biology, University of Rome Tor Vergata, Rome, Italy; ^2^IRCCS Fondazione Santa Lucia, Rome, Italy; ^3^IUF – Leibniz Research Institute for Environmental Medicine, Düsseldorf, Germany; ^4^Medical Faculty, Institute of Clinical Chemistry and Laboratory Diagnostic, Heinrich Heine University of Düsseldorf, Düsseldorf, Germany; ^5^Department of Molecular Medicine and Medical Biotechnology, University of Naples Federico II, Naples, Italy

**Keywords:** senescence, glucose metabolism, lipid metabolism, mitochondria, oxidative stress, aminoacid metabolism, signaling

Aging is a dynamic and irreversible physiological process, which leads to the progressive decline of biological functions involving all tissues and organs, due to a variety of endogenous and environmental factors. Metabolic changes are some of the hallmarks of aging and the alteration of metabolic pathways may accelerate the process and the onset of age-related diseases.

The metabolic bases of aging are lesser-known and far from being explained. There is evidence, however, that points to the role of mitochondria as key modulators of the aging process. These organelles are involved in a plethora of biological functions that affect aging both directly and indirectly. On the one hand, aging is characterized by bioenergetic failure and is accompanied by the impairment of mitochondrial dynamics and mitochondrial quality control pathways, leading to excessive production of reactive oxygen species (ROS), which are harmful to cell proteins, lipids, and DNA. On the other hand, partial reduction of mitochondrial function, as well as mild oxidative stress, have been shown to, paradoxically, promote health span across species, indicating that mitochondrial activity must be finely tuned for optimization of the aging process.

Among other organs, the brain is the most sensitive to the aging process because it requires a high amount of energy. In this Research Topic, Messina et al. comprehensively review current knowledge about the involvement of mitochondria in age-related brain dysfunctions (e.g., dementia and neurodegenerative diseases), focusing on molecular mechanisms assuring mitonuclear communications, the integrity of the mitochondrial lattice, and the cross-talk with other cellular organelles. They describe how such mechanisms become dysfunctional and affect neuronal plasticity and long-term memory storage.

The brain is a unique organ with a highly heterogeneous cellular composition and a more complex bioenergetic system than peripheral tissues. Such complexity derives not only from the variety of the residing cell types but also from the intricate network of their cellular communications. In the context of brain aging, Qi et al. summarize the different metabolic phenotypes of brain cells and describe how their metabolism is mutually modulated to maintain brain bioenergetic homeostasis. In particular, they focus on the astrocyte-neuron metabolic interactions through the lactate shuttle and the coordination of the lipid metabolism.

Cellular senescence is a process characterized by a stable and irreversible cell growth arrest and represents another well-established hallmark of the aging process. Environmental challenges such as ionizing radiation induce cellular senescence and accelerate aging. For this reason, in recent years particular attention has been given to the study of the effects of space missions on astronauts as they are exposed to cosmic rays and are more prone to develop many age-related diseases (e.g., cataract, heart and brain diseases). In this regard, Giovanetti et al. describe the main sources of radiation in space and their deleterious effects and highlight the importance of considering space flights as a precious opportunity to study the mechanisms underlying aging in humans.

Mounting evidence shows that senescent endothelial cells (ECs) can play a role in instigating the morphological and biochemical changes that accompany the vascular dysfunction typical of many age-related diseases including cardiovascular diseases (CVD). After giving a comprehensive review of the EC metabolism and metabolic reprogramming in cellular senescence, in their review, Sabbatinelli et al. emphasize that the metabolic changes occurring in senescent ECs are critical for the setting of pro-inflammatory states characterizing CVD diseases and that a deeper understanding of the factors involved in the metabolic reshaping of ECs is pivotal for finding effective therapeutic or preventive strategies to treat age-related diseases. Nitti et al. provide an up to date and critical overview of the molecular pathways by which heme oxygenase 1 (HMOX1), an enzyme involved in heme catabolism, and bilirubin, the heme degradation product, protect vascular integrity. In particular, due to these antioxidant and anti-inflammatory roles, the authors underline the importance of moderately increasing the plasma concentration of bilirubin to counteract CVD diseases. Senescent cardiomyocytes show decreased NAD^+^ levels and an increase of senescent cardiomyocytes positively correlates with cardiac diseases. Wang L.-F. et al. report that CD38, the major hydrolase for degradation of NAD^+^, is causally involved in the senescence program as this enzyme is increased in senescent cardiomyocytes and its ablation alleviates the _D_-galactose-induced myocardial cell senescence and oxidative stress by the activation of the NAD^+^/Siruin1 signaling pathway.

Brinkmann et al. and Faienza et al. focus their attention on the other two proteins that seem to play a key role in aging pathophysiology by coordinating the communication between the nucleus and mitochondria. In particular, Brinkmann et al. provide a systematic review of the existing literature about the Aryl-hydrocarbon Receptor (AhR), an evolutionarily conserved transcription factor involved in the regulation of biological responses to environmental planar aromatic hydrocarbons (e.g., dioxin, flavonoids), which was recently shown by the authors to have pro-aging effects across species. In their review, they try to reconcile the contradictory studies indicating either its pro- or anti-aging effects by a possible AhR-mitochondrial cross-talk to shed light on this factor as a means of developing anti-aging strategies. In their opinion paper, Faienza et al. draw the attention to TRAP1, a mitochondrial chaperone that is a member of the heat shock protein 90 family, involved in the maintenance of mitochondrial homeostasis. Advances in the comprehension of the post-translational modifications (i.e., *S*-nitrosylation) orchestrating TRAP1 activity and its effect on gene expression and mitochondrial physiology with relevance to aging and age-related diseases are described in detail.

In their original paper, Wang J. et al. give evidence of the involvement of altered branched-chain amino acid (BCAA) catabolism in dysfunctional glycemic control, which is typical of type 2 diabetes. By using mice with ablation of mitochondrial protein phosphatase 2C, which is involved in the BCAA degradation pathway, they show the occurrence of mild BCAA catabolic defects leading to a decrease in body weight and better tolerance of glucose in lean, but not in normal or obese animals, mainly due to amelioration of liver glucose metabolism.

The liver is a complex metabolic organ that is fundamental for maintaining metabolic health by regulating systemic lipid and glucose metabolism and by protecting from xenobiotic and endobiotic stress. For this reason, the alteration of liver function during aging increases our susceptibility to age-related diseases. Carotti et al. provide original contributions to this Research Topic, obtained both from experimental models and patients affected by non-alcoholic fatty liver disease. The authors show that liver steatosis is caused by the impairment of lipid degradation through lipophagy, which occurs to an extent that is tightly correlated with disease severity and progression.

## Author Contributions

All authors listed have made a substantial, direct and intellectual contribution to the work, and approved it for publication.

## Conflict of Interest

The authors declare that the research was conducted in the absence of any commercial or financial relationships that could be construed as a potential conflict of interest.

